# Microwave-Assisted Extraction of Bioactive Compounds from Lentil Wastes: Antioxidant Activity Evaluation and Metabolomic Characterization

**DOI:** 10.3390/molecules27217471

**Published:** 2022-11-02

**Authors:** Maria Maddalena Cavalluzzi, Antonella Lamonaca, Natalie Paola Rotondo, Daniela Valeria Miniero, Marilena Muraglia, Paola Gabriele, Filomena Corbo, Annalisa De Palma, Roberta Budriesi, Elisabetta De Angelis, Linda Monaci, Giovanni Lentini

**Affiliations:** 1 Department of Pharmacy—Drug Sciences, University Aldo Moro-Bari, Via Orabona 4, 70126 Bari, Italy; 2 Institute of Sciences of Food Production, National Research Council of Italy (CNR-ISPA), Via Amendola 122/O, 70126 Bari, Italy; 3 Department of Soil, Plant and Food Sciences, University Aldo Moro-Bari, Via Orabona 4, 70126 Bari, Italy; 4 Department of Biosciences, Biotechnologies and Biopharmaceutics, University Aldo Moro-Bari, Via Orabona 4, 70126 Bari, Italy; 5 Department of Pharmacy and Biotechnology, Food Chemistry and Nutraceutical Lab, Alma Mater Studiorum-University of Bologna, 40126 Bologna, Italy

**Keywords:** lentil hulls, microwave-assisted extraction, circular economy, antioxidant activity, liquid chromatography-high resolution mass spectrometry

## Abstract

The recovery of industrial by-products is part of the zero-waste circular economy. Lentil seed coats are generally considered to be a waste by-product. However, this low-value by-product is rich in bioactive compounds and may be considered an eco-friendly source of health-promoting phytochemicals. For the first time, a sustainable microwave-assisted extraction technique was applied, and a solvent screening was carried out to enhance the bioactive compound content and the antioxidant activity of green and red lentil hull extracts. With respect to green lentil hull extracts that were obtained with different solvents, the aqueous extract of the red lentil seed coats showed the highest total phenolic and total flavonoid content (TPC = 28.3 ± 0.1 mg GAE/g dry weight, TFC = 1.89 ± 0.01 mg CE/100 mg dry weight, respectively), as well as the highest antioxidant activity, both in terms of the free radical scavenging activity (ABTS, 39.06 ± 0.73 mg TE/g dry weight; DPPH, IC_50_ = 0.39 μg/mL) and the protection of the neuroblastoma cell line (SH-SY5Y, IC_50_ = 10.1 ± 0.6 μg/mL), the latter of which has never been investigated so far. Furthermore, a metabolite discovery analysis was for the first time performed on the aqueous extracts of both cultivars using an HPLC separation which was coupled with an Orbitrap-based high-Resolution Mass Spectrometry technique.

## 1. Introduction

Reactive oxygen species (ROS) are highly reactive, oxygen-containing molecules that are implicated in several diseases and disorders [1] since they can cause impaired antioxidant status and oxidative stress in the human body [2]. Antioxidants act as protective factors against oxidative damage as they are able to interrupt or delay oxidation by affecting the initiation or propagation steps of the oxidizing chain reactions [3], thus reducing, for example, the incidence of cardiovascular diseases and cancer [4]. Recently, the food industry has raised important questions about the effects of the prolonged consumption of synthetic antioxidants on human health [5] with a potential risk of carcinogenicity being associated with their consumption [6,7]. Therefore, plant-derived natural antioxidants have recently attracted considerable interest because of their potential nutritional and therapeutic value. It is well known that natural phenolic antioxidants can scavenge reactive oxygen and nitrogen species (RONS) thereby preventing the onset of oxidative diseases in the human body [8,9]. Moreover, epidemiological studies highlighted a positive correlation between the consumption of phenolic-rich foods and a decrease in several chronic diseases [10,11]. In the last few decades, from a circular economy perspective, there has been a growing interest in the recovery of antioxidants from by-products and agro-industrial wastes such as peels, kernels, seeds, and pomaces which are often discarded [12,13,14], thus causing an environmental problem.

Legume seed coats, which are commonly referred to as hulls, have been extensively investigated since they are rich sources of polyphenolics and natural antioxidants [15]. Lentils (*Lens culinaris* L.) are small edible legumes which have been cultivated all over the world for centuries [16,17]; they contain high levels of natural antioxidants [18,19]—such as phenolic acids, flavanols, saponins, phytic acid, and condensed tannins [20,21]—besides them being an excellent source of micro- and macronutrients. In particular, the bioactive compounds are mainly concentrated in the hull portion [22,23], the outermost part of the legume seeds which is obtained as processing waste from the decortication process, and it is normally discarded. Therefore, the recovery of these high-added-value components from lentil seed coats has become the subject of growing interest to reduce the environmental impact that is caused by this agro-industrial waste [24]. To the best of our knowledge, only a few studies report on the extraction of bioactive compounds from lentil hulls, and ultrasound-assisted extraction is the only emerging extraction technique that has been applied so far.

Based on our previous experience with the microwave-assisted extraction (MAE) of secondary metabolites from different plant matrices [25,26,27], we aimed to investigate for the first time the effect of microwaves on the extraction of antioxidant compounds from lentil hulls while using different green solvents to guarantee an eco-friendly and sustainable extraction procedure. Our investigation leads to the selection of the best extraction solvent. The antioxidant activity of the extracts that were obtained from two different lentil cultivars was evaluated for the first time on cell lines, as well as through the usually adopted chemical assays. Finally, after having assessed the most active extracts on the cell lines, the chemical profile and identification of the major metabolites that are contained therein were provided through liquid chromatography–high-resolution mass spectrometry (LC-HRMS). These cultivars were for the first time investigated through metabolite discovery analysis using HPLC separation coupled with an Orbitrap based high Resolution Mass spectrometry, which was followed by an informatic data treatment by means of dedicated software for the compound identification.

## 2. Results and Discussion

### 2.1. Evaluation of the Microwave Irradiation Effect on the Extraction of Lentil Bioactive Compounds Endowed with Antioxidant Activity and Solvent Screening

Microwave-assisted extraction (MAE) was chosen as it is an efficient and innovative green methodology with several advantages over the conventional extraction techniques, such as a higher extraction rate and therefore a shortened extraction time, a reduced solvent consumption, and a lower energy consumption [28,29]. Furthermore, high phytochemical extraction yields are often ensured due to the shorter extraction time that reduces the risk of their decomposition and oxidation [30], as we demonstrated in our previous works [25,26]. To ensure that this was a more eco-friendly procedure, our intention was also to use green extraction solvents, unlike what has been reported in the literature so far. Methanol and methanol-acetone mixtures [31,32,33,34,35] are the solvents predominantly used for the extraction of bioactive compounds from lentil hulls, but it is known that methanol is highly toxic, highly volatile, inflammable, and is still considered to be nonrenewable [36]. Starting from these considerations, ethyl acetate (EtOAc) was at first chosen as a green solvent [37], and both the lentil hulls and whole seeds of the Eston green cv. were investigated. For the sake of clarity, since no acidic or basic conditions were used, only the soluble polyphenols were extracted at this early stage of our study. The antioxidant activity of the extracts that were obtained under microwave irradiation—at 80 °C for 5 min with a solid/liquid ratio of 200 mg/mL—was evaluated on the cell lines through the 2′,7′-dichlorodihydrofluorescein-diacetate (H_2_DCF-DA) assay, and the results are reported in Table 1 (entries 1 and 2). In particular, neuroblastoma cells (SH-SY5Y) were chosen, first of all because the evidence supports the role of increased oxidative stress in the pathogenesis of neuroblastoma [38], but also because oxidative stress is a key factor in neuronal cell death in neurodegenerative diseases [39,40], so a reduction in the oxidative stress would potentially prevent neurodegeneration.

**Figure 1:**
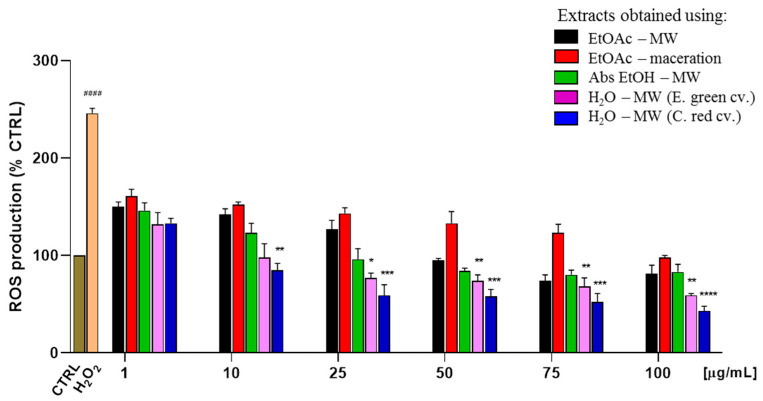
Antioxidant effects of extracts on intracellular ROS production by H_2_DCF-DA assay. SH-SY5Y cells were treated with different concentrations of extracts (0–100 μg/mL) in the presence of a toxic H_2_O_2_ stimulus (200 μM). ROS production was evaluated by measuring the fluorescent intensity of control cells that were treated only with H_2_DCF (CTRL). Quercetin at 25 μg/mL (QRC) was used as the positive control, and 89% ROS production inhibition was observed. Statistical significance was calculated using a one-way analysis of variance (ANOVA) which was followed by the Dunnett’s test (Graph Pad Prism version 5): * *p* < 0.1, ** *p* < 0.01, *** *p* < 0.001, **** *p* < 0.0001 referred to H_2_O_2_ alone; #### *p* < 0.0001 referred to CTRL.

According to what is reported in the literature for other cultivars, our study confirmed that the antioxidant content of lentil hull Eston green cv. is higher than that which was found in the seed, with their corresponding IC_50_ values being 87 ± 2 μg/mL and >100 μg/mL, respectively. This is due to the presence of bioactive molecules on the outer surface of the seed which are used by the plant as protection against predators. To have more insights into the most abundant compounds characterizing this fraction, an in-depth investigation was carried out on the lentil hull as this is a waste product of the lentil decortication process. To evaluate the effects of microwave irradiation on the extraction efficiency, MAE was compared with a conventional extraction process, and according to our findings, the former demonstrated about a 1.5-times higher extraction power than the maceration (entries 2 and 3, Table 1). This result may be explained by assuming that the rapid rise in the temperature that was reached through microwave irradiation reduces the viscosity of the extract, thus promoting the solubility of the target components. In parallel, no degradation of the bioactive compounds occurred at the higher temperatures that were reached while they were under microwave irradiation when the short extraction times were ensured. Unfortunately, the cytotoxic IC_50_ value of the most active extract (entry 2) was very close to that which was obtained for the antioxidant activity (110 ± 3 and 87 ± 2 μg/mL, respectively), therefore, the effect of different extraction solvents was evaluated. Green solvents with different polarity degrees were chosen: cyclopentyl methyl ether (CPME), absolute ethanol (abs EtOH), and water. It is noteworthy that a greater dilution was required when water was used as the solvent (50 mg/mL as solid/liquid ratio) since an excessive gelling of the solution was observed at the higher concentration reached for the organic solvents, thus making it impossible to remove the exhausted matrix at the end of the extraction process. The three so-obtained extracts were then tested at three different concentrations (1, 10, and 50 μg/mL) and good results were observed except for the CPME extract (data not shown). Therefore, the IC_50_ values of both the hydroalcoholic and aqueous extracts were determined, and as reported in Figure 1 and Table 1 (entries 4 and 5), water gave the most active extract with the IC_50_ value being the lowest among all of the extracts and it being eight-fold lower than its cytotoxic concentration. The different results that were obtained should derive from the different nature of the secondary metabolites that were recovered using solvents with increasing polarity: water proved to be the best solvent, both in terms of the extraction yield and the antioxidant activity displayed by the extracts, probably because the water-soluble phenolic acids and flavonoids, as well as their glycosides, were more extracted. To explore the possibility of obtaining an extract with greater antioxidant potency, a different lentil variety which was probably endowed with a higher content of antioxidant compounds due to its dark color—namely Crimson red—was subjected to water extraction. As it is known the greater antioxidant activity of lentil hulls compared to the seeds [22,23,31,35], the extraction was carried out only on Crimson red hulls. As reported in Table 1 (entry 6), not only did the red lentil hull extract not show cytotoxicity at the highest tested concentration, but it was also about 2.5-fold more potent than the Eston green cv. was, thus confirming that the red color lentils are in the high-antioxidant foods category [41]. It is noteworthy that this interesting result was achieved with water, the quintessential as well as the cheapest and most easily available green solvent which meets the ecological need to limit the use of toxic organic solvents. Having also used a green extraction technique, both the possible environmental toxicity and the cost that is associated with the extraction procedure have been reduced.

Summing up the results of our study, the aqueous extracts of the two investigated lentil hull varieties showed the highest antioxidant activity on the SH-SY5Y cellular line, with Crimson red extract being more effective than the Eston green one.

### 2.2. Chemical Assays for the Measurement of the Antioxidant Activity

The high antioxidant potential of phenolic compounds being known, their quantification is a common practice in food research, and the Folin–Ciocalteu assay is the most commonly used procedure to determine the total phenolic content (TPC) of these extracts. However, an overestimation of the polyphenol content could occur during the assay since the Folin–Ciocalteu reagent interacts with other non-phenolic substances, such as water-soluble reducing sugars. Therefore, the aluminum chloride colorimetric assay was also carried out to determine the total flavonoid content (TFC), thus providing us with more detailed information. As reported in Table 2, the total phenolic content in Crimson red was about 4-fold higher than it was in the Eston green variety, with their values being 28.3 ± 0.1 and 7.88 ± 0.1 mg gallic acid equivalents (GAE)/g dry weight, respectively. A more pronounced difference between the two cultivars was observed in terms of the total flavonoid content (TFC) having obtained 1.89 mg of catechin (CE)/100 mg dry weight for Crimson red which is more than 20-fold higher than 0.08 mg CE/100 mg of Eston green dry weight.

To chemically evaluate the in vitro antioxidant activity of our extracts, we chose ABTS and DPPH assays which are among the most widely used ones for studying vegetable materials. In both of the methods, the radical scavenging activity of the antioxidant-containing sample is determined in terms of the absorbance changes of the colored and stable ABTS+ or DPPH· radicals, respectively. In agreement with the different content of antioxidant bioactive compounds that were observed in the two previous assays, the results that were obtained in the ABTS assay demonstrated that the antioxidant activity of Crimson red extract was 10-fold higher than that of the Eston green extract in terms of Trolox equivalent (TE)/g dry weight. Analogously, the concentration of Crimson red necessary for a 50% reduction in the DPPH radical was lower than that which was observed for Eston green, thus confirming the higher antioxidant capacity of the richer phenols and flavonoids of Crimson red extract.

It is noteworthy that our study highlights the extraction efficiency of MAE as herein we describe for the first time an extraction procedure of lentil seed coats which was carried out in only 5 min rather than from tens of minutes to hours, thus ensuring low energy costs as well as the reduced possibility of thermal degradation occurring given the reduced exposure time of the bioactive compounds to heat. Unfortunately, the obtained results cannot be compared with the ones in the literature as sometimes the used variety is not specified, different assays have been carried out, or different units of measurement have been used to express the results.

### 2.3. Metabolites Profiling of Lentil Hulls

To obtain more insights into specific compounds, accounting for the biological activity values that were found for Eston green and Crimson red lentil hulls, their aqueous extracts were submitted to liquid chromatography/tandem mass spectrometry (LC-MS/MS) untargeted analysis for the metabolites profiling. The MS spectra were then processed using the commercial software Compound Discoverer v. 3.3.1.111 SP1 (Thermo Fisher, Bremen, Germany), and identification was accomplished by activating the Chem Spider and mzCloud nodes. The identification of such putative compounds can be performed at two different stringency levels, namely medium or more advanced confidential identification, depending on the degree of information that is provided, i.e., whether it is based on accurate masses of the only precursor ions (with a mass tolerance lower than 3 ppm), or if there is a recognition of the most intense fragment ions which are deposited in mzCloud databases compiling experimental fragment ions that have been validated by other analysts. Thanks to the intrinsic characteristics of these two identification tools, it was possible to identify a multitude of compounds according to the “level IIa” (probable structure = more advanced) and “level III” (putatively characterized = medium) criteria which were set out by Metabolomic Standard Initiative [42,43]. Each molecule was searched for in the PubChem online database to gather information about their class and their putative function.

**Figure 2:**
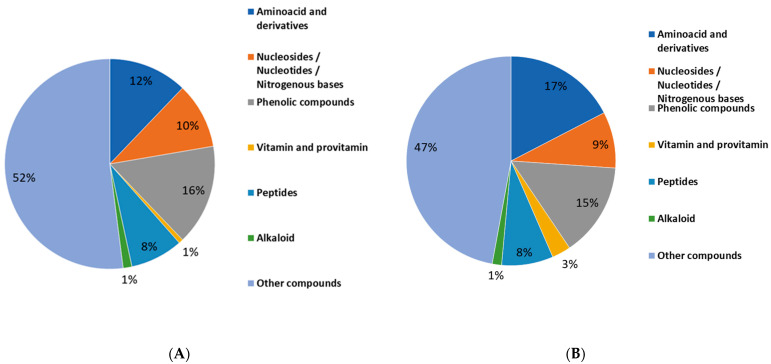
Percentage composition of the most relevant class of compounds which were identified in hulls of Eston green (panel **A**) and Crimson red (panel **B**) lentils according to the results that were retrieved using Compound Discoverer software.

As for the green lentil hulls, 2745 features were retrieved by the software, then reduced to 142 after a visual validation which was performed by the operator. Specifically, 109 compounds were selected from ChemSpider results, while 133 molecules were obtained from the mzCloud search. On the contrary, 3474 features were displayed for the red lentil hulls, among which only 116 and 140 compounds were validated from Chem Spider and mzCloud results, respectively. In Table 3, the most relevant molecules which were identified by the Chem Spider and mzCloud searches of the lentil hulls of cv. Eston green and Crimson red are presented, while in Figure 2 (panels A and B), the percentage composition of the identified classes that are putatively endowed with biological activity is attributed to common nutrients as shown.

At a glance, the metabolic profiles of the lentil hulls that are illustrated in Figure 2 (panels A and B) reveal that this by-product is generally rich in nutritional and bioactive compounds, and indeed, good percentages of molecules such as phenolic compounds, nucleosides/nucleotides/nitrogenous bases, peptides, vitamins, amino acids, and derivatives were found in the green and red samples.

**Figure 3:**
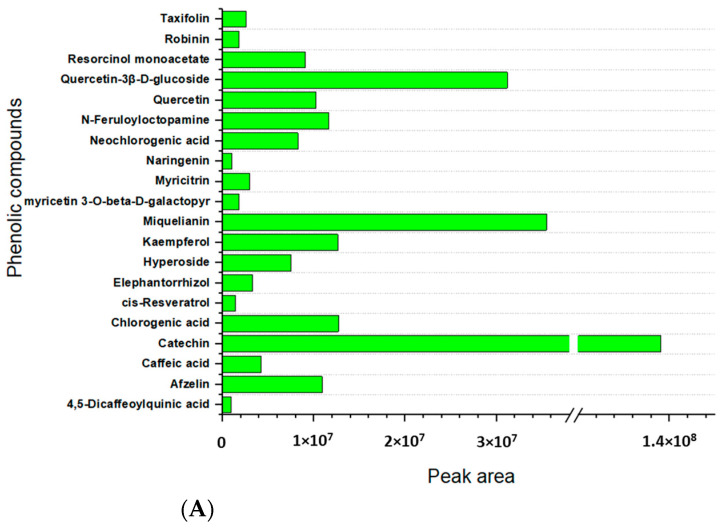

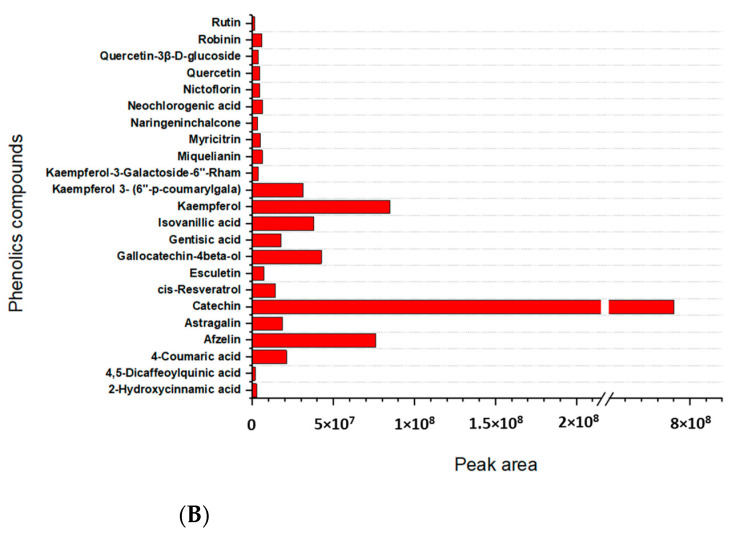
Comparative analysis based on peak areas that were calculated for phenolic compounds that were found in Eston green (panel **A**) and Crimson red (panel **B**) hulls. The peak area of each phenol was retrieved using the software Compound Discoverer 3.3.

Among the bioactive compounds, a consistent fraction of phenolic compounds was observed in the hulls of both the green and red variety, accounting for 15% and 16%, respectively, although the specific composition slightly differs between the two samples (see Table 3). It is worth noting that the percentage composition of the classes that are shown in Figure 3, which refers to a list of the identified compounds, do not to be ignore that other molecules could have been present in the aqueous extracts of the two samples, however they may have escaped the LC/MS analysis for having too low an intensity or they could have been excluded because they do not fulfil the stringent filters that were applied during the identification process. This is the case for the phenolics, for which the assay on the total content (TCP) revealed that Crimson red was approximately 4-fold richer in phenolics when it was compared to Eston green, while a similar percentage content of this class was displayed when the identified phenols were compared (Figure 3).

Regarding the identification results, many of the detected compounds with a proven antioxidant capacity among which catechin, miquelianin, neochlorogenic acid, kaempferol, myricitrin, quercetin, quercetin-3β-D-glucoside, *cis*-resveratrol, and robinin—were found in both of the varieties. On the contrary, some of the phenolics displayed a differential pattern; while caffeic acid, chlorogenic acid, elephantorrhizol, hyperoside, myricetin 3-*O*-beta-D-galactopyranoside, naringenin, *N*-feruloyloctopamine, resorcinol monoacetate, and taxifolin were extracted only from the green lentil hulls, 2-hydroxycinnamic acid, 4-coumaric acid, astragalin, esculetin, gallocatechin-4beta-ol, gentisic acid, isovanillic acid, kaempferol derivatives, naringeninchalcone, nictoflorin and rutin were observed only in the red cv. Moreover, both of the varieties contain, although in low percentages, trigonelline and sinapine alkaloids which are molecules that are renowned for their favorable biological activities [44].

Many papers in the literature reported that pulse hulls significantly contribute to the phytochemical content of whole seeds [31,45] thanks to their high levels of polyphenols or alkaloids, which are molecules that are known for their beneficial impact on human health [44]. For example, the total phenolic content of the lentils hulls was reported to be 3–8-fold higher than that of the whole seed [46], and the phenolic composition was found more variegated than the cotyledon counterpart [47,48,49]. In 2020, Sun and co-workers studied the main hydrophilic and lipophilic phytochemicals of three lentils cv., including Eston green, and they assayed in vitro the antioxidant capacities of each of the lentil extracts [33]. In general, a high content of free extractable phenolics (phenolic acids, flavonols, and procyanidins), with the procyanidins being the major group of the extractable free phenolics, was found in the lentil hulls together with a considerable amount of conjugated and bound forms of phenolics. Interestingly, Sun and co-workers described that green lentil hulls show higher total and individual phenolic contents than red lentil hulls (Red cv.) do, which directly translated to a higher antioxidant capability of the former lentil in their work. Conversely to that which was observed by Sun et al., we found that there was a comparable phenolics content in the green and red lentil hulls, although a slightly different composition was displayed for the two samples. In Figure 3, a comparative analysis based on the peak area that was calculated for each of the phenol compounds that were detected in green (panel A) and red (panel B) lentil hulls, respectively, was reported.

**Figure 4:**
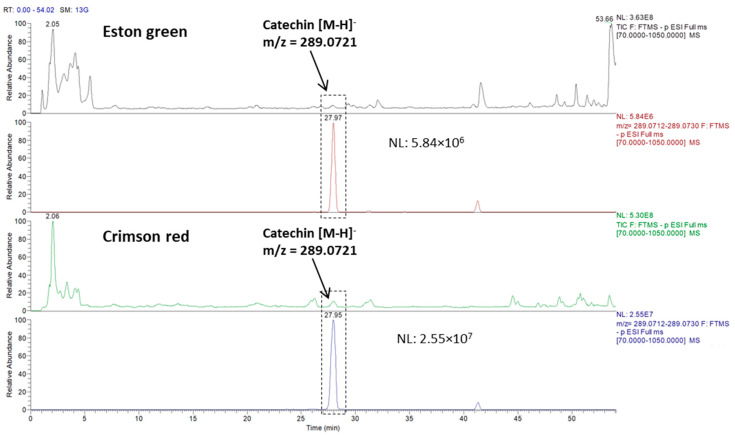
Comparison of TIC chromatograms referred to Eston green and Crimson red lentil hulls which were obtained by running the LC/MS instrument in FullMS/ddA acquisition mode along with the respective XIC of catechin.

As it appears in the graphs, a high catechin content was observed in both of the samples; the graphs show values in the peak area that are four times higher than the miquelianin, which is the second most abundant phenol that was found in the Eston green variety, and it was nine times higher when it was compared to kaempferol in the Crimson red sample. Interestingly, the red lentil hulls show a catechin content (based on peak area) that is approximately 6 times higher compared to the green counterpart. Figure 4 pictures an overlay of chromatograms that are relevant to two total ion current (TIC) traces which are referred to as the Eston green and Crimson red samples, and these were analyzed in fullMS/data-dependent acquisition (FullMS/ddA) mode and the extracted-ion chromatogram (XIC) traces which were filtered for the precursor ion of catechin. As for the other phenols, the Eston Green lentil hulls (panel A) showed a high peak area for miquelianin, which was followed by quercetin-3β-D-glucoside and afzelin which had a peak area value that was very close to that which was found for chlorogenic acid, kaempferol, and *N*-feruloyloctopamine. Other phenols showed lower peak areas, thus accounting for a lower content of them. As for the red lentil (panel B), kaempferol was the predominant phenolic compound after catechin, followed by afzelin and gallocatechin-4beta-ol; this last one shows a peak area value that is quite similar to that for kaempferol 3-(6″-*p*-coumarylgala) and isovanillic acid. Lower peak area values were displayed for the other phenols.

In general, when we are comparing both of the lentil cultivars, a greater concentration of free polyphenols was evident in the black lentil extracts compared to the most abundant flavonoid glycosides in the green one. Oddly, no anthocyanins have ever been detected, although they are usually described as being major components of lentil hull extracts. On the other hand, we expected to find them at least in the red lentils since they are typically responsible for the dark color of lentil seeds. We speculate that microwave irradiation may have caused the degradation of the anthocyanins, as reported in the literature for fruits and vegetables [50]. This is why the Design of Experiments (DoE) approach will be carried out to optimize the extraction process with the aim of recovering the greatest possible content of bioactive compounds, including anthocyanins.

The different distribution of phenolic compounds found between our green and red samples as well as between our results and those previously reported [32,33] could be explained by taking into account that the final content of the phenolics of lentils is strongly influenced by the genotype and the environmental conditions of growth of the sample, as demonstrated by Rochfort et al. in 2019. Specifically, the authors profiled, by using a ^1^H NMR technique, 14 different genotypes of red and green lentils that were grown in different soil and environmental conditions, revealing that both the genotypic and environmental factors deeply affected the accumulation of healthy metabolites of the lentils. In particular, the hulls were found rich in polyphenolics, with levels of them that were variable based on the genotype. Moreover, their composition and concentration showed to be strongly influenced by abiotic effects [51].

Besides the different distribution, the phenolic compounds that were identified in both the green and red varieties also showed different contents (calculated by peak area estimation) that could be directly correlated to the high biological activity that was observed on the SH-SY5Y cellular line. In a very recent paper, the digestive products of Laird lentil hulls were explored for their anti-inflammatory mechanism by using the NF-κB and Keap1-Nrf2 signaling pathways in the HT-29 cell model [52]. The authors found a total of 27 polyphenols and five nonphenolic constituents and among them, catechin glucoside, kaempferol tetraglucoside, procyanidin dimer, and dihydroxybenzoic acid-*O*-dipentoside were the main polyphenols in the digestive products. These digestive products could reduce the inflammatory mediators and exert anti-inflammatory activity by inhibiting NF-κB and activating the Keap1-Nrf2 signaling pathways, and there was crosstalk between them, which was a mutual inhibition effect [52]. In the same year, the same authors reported that pea hull polyphenols could be continuously released and absorbed upon digestion, thus playing a positive role in protecting the intestinal barrier and anti-inflammatory activity, with the kaempferol trihexoside being the compound that was highly absorbed and transported in digestive products [53]. The role of polyphenols in the anti-inflammatory capacity of lentil hulls was reported also by Peng et al., by testing their digestive products on the Caco-2 cell monolayer and Caco-2/RAW264.7. In this case, the protocatechuic acid glycoside derivative was found to have the highest content in the lentil hulls digestive product [54]. These investigations suggest the key role of the phenolics that are contained in the lentil hulls on the beneficial effects that are exerted on biological systems. Therefore, it could be speculated that the high content of phenols that are found in the Eston Green and Crimson Red lentil hulls significantly contribute to the biological activity that is observed in the cell lines that were tested. Moreover, the higher content of kaempferol and catechin which was found in the Crimson red variety with respect to the amount of those that were found in the green could be directly involved in the higher antioxidant activity that is observed and attributed to this variety.

It is worth to be noted that a deepened investigation that should be carried out on the selected phenolic compounds for quantitative purposes could help research to achieve in the future more accurate characterization and quantification of the single phenolic compounds which should be unambiguously correlated to the biological activity that is displayed.

In addition to the phenolic compounds, the Eston green and Crimson red lentil hulls were also observed to contain alkaloid compounds, such as trigonelline and sinapine, although at low concentrations with respect to the total compounds that were identified (2%). Interestingly, although they are historically referred to as “anti-nutritional factors”, trigonelline and sinapine are molecules that are renowned for their favorable biological activities. For example, trigonelline is involved in the protection of the heart and liver and it has beneficial effects against hyperglycemia, hypercholesterolemia, nervous and hormonal disorders, and cancers [55], while sinapine is important for its antioxidant and radio-protective activities [56]. The presence of these compounds confirms once again the bioactive potential of lentil hulls and their potential benefits to human health.

Finally, our results point out that green and red lentil hulls are also a good source of nutritional compounds; indeed a good content of amino acids and derivatives, as well as nucleosides/nucleotides/nitrogenous bases and vitamins/provitamins were observed in both of the samples that were analyzed (Table 3). It is noteworthy that 8% of all the metabolites which were revealed by the bioinformatic search were attributed to small peptides, such as di-, tri-, and tetra-peptides. Since the increasing interest of the consumers and food industries for food-derived bioactive peptides (BP), all of the peptide sequences that were retrieved by the software for the green and red lentil hulls were searched for in the online freely-accessible BIOPEP-UWM^TM^ database [57,58], which is a popular tool used for identifying bioactive peptides, especially those derived from foods and being constituents of diets that prevent the development of chronic diseases. The results are listed in Table 4. In detail, four peptide sequences that were extracted from the green and red lentil hulls exactly matched with some of the bioactive peptides of the BIOPEP-UWM^TM^ platform, namely AP, GSH, NYY, and PPPS, whereby AP and GSH were found in the green variety, while NYY and PPPS were found in the red one. Other peptides, such as QYW and EWE were recognized as substrings of four and two different bioactive peptides, respectively. BP have been demonstrated to have a positive impact on body functions or conditions, and they may influence health [59]. Based on their mode of action, they can be classified as antimicrobial, antithrombotic, antihypertensive, opioid, immunomodulatory, mineral binding, and antioxidant types [60]. In particular, of bioactive peptides that were extracted from the lentil hulls, NYY and GSH are described to be involved in the reduction of oxidative stress, working as antioxidant agents [61,62], while AP and PPPS act as enzymatic inhibitors [63,64]. GSH was recognized also as Angiotensin Converting Enzyme (ACE) inhibitor [65].

The findings presented herein highlight that lentil hulls could be considered an interesting source for the human diet as they are rich in phytochemicals and nutritional compounds, thus promoting beneficial effects on human health.

## 3. Materials and Methods

### 3.1. Lentil Materials

Lentils seeds and hulls (*Lens culinaris* var. Eston green or Crimson red), harvested in 2021 from Piana Cardone in Agro di Genzano (Lucania cultivar, Basilicata, Italy), were kindly provided by Agricola Piana d’oro. In particular, lentil seeds were ground to powder using a stone mill (Brugnoni s.r.l., Treia (MC)) and passed through standard sieves. The powders were stored in the dark at 4 °C before use. The lentil seed coat was separated from the cotyledon using a semi-industrial husker (Baragioli mechanical workshop of Baragioli Marco & c. s.n.c., Vercelli) at a rotor speed of 7.5 KW power. The obtained hull was collected, vacuum-packed in plastic bags, and stored before use.

### 3.2. Chemicals

Solvents used for extractions were purchased from Sigma-Aldrich (Milan, Italy).

Assay on neuroblastoma cell line SH-SY5Y: Dulbecco’s modified Eagle’s medium high glucose (DMEM), fetal bovine serum (FBS), penicillin, l-glutamine, streptomycin and trypsin were purchased from Euroclone (Italy); 3-(4,5-dimethylthiazol-2-yl)-2,5-diphenyltetrazolium bromide (MTT) and H2DCFDA (H2-DCF, DCF) were provided from Invitrogen (Thermo Fisher Scientific, Waltham, Massachusetts, US); quercetin (QRC) and hydrogen peroxide 30% *v/v* (H_2_O_2_) were purchased from Sigma-Aldrich (Milan, Italy). SH-SY5Y cells were purchased from the American Type Culture Collection (ATCC), Rockville, MD, USA.

Chemical assays: MeOH, Folin–Ciocalteu reagent, gallic acid (GA), sodium carbonate, catechin (CE), aluminum chloride, sodium-potassium tartrate, 2,2-diphenyl-1-picrylhydrazyl (DPPH) and 6-hydroxy-2,5,7,8-tetramethyl chromane -2-carboxylic acid (Trolox) were purchased from Sigma-Aldrich (Milan, Italy). 2,20-Azinobis(3-ethylbenzothiazoline-6-sulphonic acid)diammonium salt (ABTS) was purchased from Alfa Aesar (Karlsruhe, Germany).

Metabolomic analysis: acetonitrile (AcN, Gold HPLC ultragradient) and methanol (MeOH, HPLC grade) were purchased from Carlo Erba Reagents (Cornaredo, Milan, Italy), while ultrapure water (H_2_O) used was produced using a Millipore Milli-Q system (Millipore, Bedford, MA, USA). Formic acid (FA, MS grade) was purchased from Fluka (Milan, Italy), while syringe filters (0.45 µm of porosity in regenerated cellulose RC) were purchased from Sartorius (Gottingem, Germania).

### 3.3. Microwave-Assisted Extraction (MAE)

A closed-system MAE was carried out at a constant temperature under continuous stirring condition in a CEM Discover Bench Mate microwave reactor that was equipped with Synergy software. The temperature was measured and controlled using a built-in infrared detector. Briefly, 400 mg of seeds or peels lentil powder in 2 mL of the appropriate solvent (100 mg/2 mL only in the case of deionized water) were irradiated using microwaves at 80 °C for 5 min. The solution was then filtered through Whatman (No. 1) filter paper, and the solvent was evaporated under reduced pressure. Water was removed by freeze-drying. The samples were stored at −20 °C until needed for analysis.

### 3.4. Conventional Extraction

A suspension of 6.0 g of hull powder in 30 mL of EtOAc was stirred at room temperature for 24 h. The solution was then filtered through Whatman (No. 1) filter paper, the solvent was evaporated under reduced pressure, and the sample (0.29 g, yield 4.8%) was stored at −20 °C until it was needed for analysis.

### 3.5. Neuroblastoma Cell-Based Assays

#### 3.5.1. Cell Viability

A human neuroblastoma cell line was cultured in Dulbecco’s modified Eagle’s medium (DMEM) high glucose that was supplemented with 10% (*v*/*v*) heat-inactivated fetal bovine serum (FBS), 2 mM L-glutamine, 100 U/mL penicillin and 100 μg/mL streptomycin at 37 °C in an atmosphere of 5% CO_2_. After their growth, the collected cells were plated at a density of 3 × 10^4^ cells/well in a 96-well plate until 70–80% confluence and then, incubated for 24 h with the extracts which were used in a range of concentrations from 1 to 100 μM to evaluate cell viability by the MTT test [3-(4,5-dimethylthiazol-2-yl)-2,5-diphenyl tetrazolium bromide] [66]. The method is based on the ability of viable cells to metabolize the water-soluble salt yellow-colored MTT by cellular oxidoreductase into a water-insoluble blue formazan product. The amount of produced formazan was proportional to the viable cells. Therefore, after incubation of the cells for 24 h at 37 °C in 5% CO_2_ with the extracts, the medium was removed, and an MTT solution in PBS 1X (0.5 mg/mL) was added in each well for 2 h. After this time, the medium was substituted by DMSO to dissolve the formazan product and absorbance values were measured at 545 nm using a multilabel plate counter Victor3 V (Perkin Elmer, Milan, Italy). Triplicate cultures were set up for each concentration used and each experiment was performed three times. All of the samples were solubilized in DMSO.

#### 3.5.2. ROS Generation and Antioxidant Effects

To evaluate the antioxidant effects of the extracts, the decrease of ROS production was tested by 2′,7′-dichlorodihydrofluorescein-diacetate H_2_DCF-DA assay.

Briefly, H_2_DCF was applied as H_2_DCF-DA, which was taken up by the cell, where unspecific esterases cleaved the lipophilic groups, thus resulting in a charged compound. Oxidation of H_2_DCF by ROS converts the molecule to DCF, which is fluorescent. The test was performed in a medium free of serum because serum contains endogenous esterases which will cleave the ester group of H_2_DCF-DA.

As previously reported [67], confluent SH-SY5Y cells were incubated for 30 min with 10 μM of H_2_DCF-DA in serum-free DMEM. After their incubation, the cells were washed with 1X PBS and incubated for an additional 30 min with 200 μM H_2_O_2_ in the presence of increasing concentrations of extracts from 0 to 100 μM at 37 °C in 5% CO_2_. QRC at 25 μg/mL and the extracts alone at 1 and 100 μM were used as standard controls. QRC significantly reduced ROS production (89% inhibition compared to the effect of toxic stimulus), while no effect was observed in the presence of extracts alone. The formation of fluorescent dichlorofluorescein (DCF) was assessed by a spectrofluorimetric analysis which was performed at an excitation wavelength of 485 nm and an emission wavelength of 528 nm using a multi-plate reader Victor3 V (Perkin Elmer, Milan, Italy).

Three independent experiments with three replicates were carried out, and the results were averaged and expressed as a percentage of ROS production referred to control cells that were treated only with H_2_DCF.

#### 3.5.3. Statistical Analysis

Graphical analysis was performed by using the GraphPad Prism 5.0 (GraphPad Software Inc., San Diego, CA, USA). and statistical analysis was carried out using one-way analysis of variance (ANOVA) which was followed by the Dunnett’s Multiple Comparison post hoc test for multiple comparison analysis. Levels of significance were reported towards the oxidative insult represented by H_2_O_2_ alone. * *p* < 0.1, ** *p* < 0.01, *** *p* < 0.001, **** *p* < 0.0001, #### *p* < 0.0001 referred to CTRL.

### 3.6. Chemical Assays

#### 3.6.1. Determination of Total Phenolic Content (TPC)

The total phenolic content (TPC) in the lentil extracts was determined by adapting the protocol that was described by Clodoveo et al. [68] in a microplate format. More specifically, the assessment of F–C reduction capacity was performed on a microplate reader (Tecan Infinite Pro 200 Microplate Reader) using spectrophotometric detection and 96-well microtiter plates (Greiner 96 Flat Bottom Transparent Polystyrene). Hence, 12.5 µL of standard gallic acid (GA) solution or diluted lentil extracts were placed in each well. Subsequently, 12.5 µL of MeOH in 50 µL of distilled water, 12.5 µL of F–C reagent and 112.5 µL of distilled water were added to each well. After 5 min, 50 µL of Na_2_CO_3_ solution (20%) was added to each well, and the mixture was incubated to react for 90 min at 30 °C. Then, the absorbance values have been registered at t λ = 700 nm. A series of GA standard solutions at different concentrations (0.025–0.25 mg/mL) was prepared and used to construct a calibration curve (y = 3.3467x + 0.0018, R^2^ = 0.9814). MeOH was used as a blank in place of the samples and standard. The TPC value was expressed as milligrams of gallic acid equivalent (GAE) per g of lentil extract (mg GAE/g of extract).

#### 3.6.2. Determination of Total Flavonoids Content (TFC)

Total flavonoids were estimated by adapting the aluminum chloride colorimetric assay reported by Rached et al. [69] in a microplate format. In detail, the total flavonoid content (TFC) in the lentil extracts was performed on a microplate reader (Tecan Infinite Pro 200 Microplate Reader) using spectrophotometric detection and 24-well microtiter plates (Greiner bio-one CELLSTAR).

In each well, 75 µL of 5% NaNO_3_ were added to 250 µL of samples/standard in 1 mL of distilled water. After 6 min, 75 µL of 10% AlCl_3_ was added. After a further 6 min, 1 mL of 4% NaOH and 100 µL of distilled water were added up to a final volume of 2.5 mL for each well. The final mixture was left at room temperature for 15 min away from light. The absorbance of the mixtures was measured at λ = 510 nm. A series of Catechin standard solutions at different concentrations (0.015–0.35 µg/mL) was prepared and used to construct a calibration curve (y = 33,764x + 0,0679, R^2^ = 0.998). Total flavonoid contents (TFC) were expressed in terms of micrograms of Catechin equivalent (CE) per 100 mg of lentil extracts (ugCE/100mg extract). The experiment was repeated three times at each concentration.

#### 3.6.3. Antioxidant Assay

##### DPPH Assay Procedure

The free radical scavenging ability of the lentil extracts was tested by adapting DPPH radical scavenging assay described by Milani et al. [25] in a microplate format. In detail, the antioxidant profile in the lentil extracts was performed on a microplate reader (Tecan Infinite Pro 200 Microplate Reader) using spectrophotometric detection and 96-well microtiter plates (Greiner 96 Flat Bottom Transparent Polystyrene).

In each well, 162.5 µL of DPPH solution (0.1 mM in MeOH) freshly prepared were added to 87.5 µL of samples in MeOH at different concentrations (0.20–125 µg/mL). The reaction mixture was incubated to react at 30 °C for 30 min. A mixture of 87.5 µL of MeOH and 162.5 µL of DPPH solution was prepared as the control sample. The absorbance of the mixture was estimated spectrophotometrically at λ = 517 nm.

The percentage of DPPH radical scavenging activity was calculated by the following equation:

% DPPH radical scavenging activity = (A_control_ − A_sample_)/A_control_ × 100 (1)

where A_control_ is the absorbance of the control and A_sample_ is the absorbance of the extract.

Antiradical curves were plotted, referring to concentration on the *x*-axis and % DPPH radical scavenging activity on the *y*-axis. Then, IC_50_ (μg/mL necessary for 50% reduction of the DPPH radical) was calculated from the graph. The experiment was repeated three times at each concentration.

##### ABTS Assay Procedure

The antioxidant activity of the lentil extracts was estimated by adapting ABTS radical cation decolorization assay according to the procedure previously reported by Milani et al. [25], with some modifications. The modified procedure was performed on a microplate reader (Tecan Infinite Pro 200 Microplate Reader) using spectrophotometric detection and 96-well microtiter plates (Greiner 96 Flat Bottom Transparent Polystyrene).

The stock solutions included 7 mM ABTS+ solution and 2.45 mM di K_2_S_2_O_8_. The working solution was then prepared by mixing the two stock solutions in equal quantities (5 mL) and allowing them to react for 16 h at room temperature in the dark. The solution was then diluted by mixing 1.4 mL ABTS+ solution with 30 mL methanol to obtain an absorbance of 0.74 ± 0.03 units at 734 nm using the spectrophotometer. ABTS + solution was daily prepared for each assay. The standard curve (y = −0.0028x + 0.5106, R^2^ = 0.992) was linear and it was in the range of 0 to 150 µg/mL Trolox.

Briefly, 10 μL of each sample/standard solution were mixed with 190 μL of ABTS+ in a 96-well microplate. The mixture was then incubated at 37 °C for 10 min before the absorbance was read at λ = 734 nm. Results are expressed in milligrams of Trolox equivalents (TE) per g of lentil extract (mg TE/g lentil extract), and they were calculated by using the following formula.

ABTS radical scavenging activity = [(A_blank_ − A_sample_)/A_blank_] × 100 (2)

The experiment was repeated three times at each concentration.

### 3.7. Metabolite Profiling of Lentil Hulls

#### 3.7.1. Sample Preparation

Lyophilized samples of lentil hulls extracts (cv. Eston green and Crimson red) that were obtained by microwave-assisted extraction with H_2_O were further dissolved in MeOH: H_2_O 80:20 (*v*/*v*) solvent to obtain a 10 mg/mL stock solution. After 5 min of vigorous shaking, samples were sonicated in an ultrasonic bath for 15 min at room temperature and 15 min at 40 °C to facilitate aggregate dissolution. Samples were further diluted in a ratio 1:2 with the mixture H_2_O:MeOH (95/5, *v*/*v*) (final matrix concentration 5 mg/mL) and filtered through a 0.45 µm syringe filter before their injection into LC/MS apparatus.

#### 3.7.2. LC-HRMS Analysis

Metabolic profiling of lentil hull samples was carried out by LC-MS/MS analysis on a benchtop Q-Exactive™ Plus Hybrid Quadrupole-Orbitrap™ High-Resolution Mass Spectrometer which was coupled with a UHPLC pump system (Thermo Fisher Scientific, Bremen, Germany). Chromatographic separation of metabolites was accomplished on an Acclaim^TM^ 120, C18 analytical column (3 µm, 120 Å, 2.1 × 150 mm, Thermo Fisher Scientific, Bremen) at a flow rate of 200 µL/min according to the following elution gradient: for 0–40 min, solvent B increased from 5% to 25%; for 40–50 min, there was a further increase of B from 25% to 50%; after 1 min, solvent B was increased to 90% and then, it kept constant for 10 min. Finally, solvent B was decreased to 5% after 1 min, and these conditions were kept constant for 18 min for column conditioning. Solvent A = H_2_O + 0.1% FA, solvent B = Methanol + 0.1% FA. The temperature of the column was kept to 25 °C along the run, the volume injection was set to 10 µL, and each sample was injected twice in MS.

Spectra were acquired both in the positive and negative ion modes in the mass range of 70–1050 *m*/*z* by running the instrument in FullMS/Data-dependent acquisition mode (FullMS/DD^2^). MS parameters were set up as follows: Full-MS event: microscan 1, resolution 70 k, AGC target 1×10^6^, maximum injection time 30 ms; dd-MS^2^ event: microscan 1, resolution 17.5 k, AGC target 1×10^5^, maximum injection time 60 ms, loop count 10, isolation window 2.0 *m*/*z*, stepped collision energy 25, 35, 55; dd settings: minimum AGC target 5×10, intensity threshold 8.3×10^2^, charge exclusion 4-8, >8, exclude isotopes on, dynamic exclusion 10 s. Source conditions were as follows: sheath gas flow rate 25, auxiliary gas flow rate 15, spray voltage 3.4 kV, capillary temperature 320 °C e S-lens RF level 55.

#### 3.7.3. Metabolite Identification

Ultrahigh-performance liquid chromatography–high-resolution mass spectrometry raw data were acquired using Xcalibur software (version 2.1, Thermo Fisher Scientific, Bremen, Germany), while peaks alignment, background subtraction and features extraction were performed using Compound Discoverer software (version 3.3.1.111 SP1, Thermo Fisher Scientific, Bremen, Germany).

The values of the critical parameters for feature extractions were the following: precursor ion deviation 2 ppm (for the positive/negative runs); maximum retention time shift 0.2 min; minimum base peak height for a peak to be retained 90,000 Arbitrary Unit (AU). Unknown compound identification and prediction of the elemental composition were accomplished by performing the Chem-Spider and mzCloud nodes searches. Mass tolerance set for the search was better than 5 ppm. Chemspider databases including the Carotenoids Database, ChEBI, ChemBank, ChEMBL, DrugBank, FDA, FooDB, LipidsMAPS, Peptides, Phenol-Explorer and PubMed ones, were queried for compound identification. While the Endogenous Metabolites, Natural Products/Medicines, Natural Toxins, Pesticides/Herbicides and Small Molecule Chemicals databases were interrogated for mzCloud-based identification. As for molecule identification, the criteria that were set out by the Metabolomic Standard Initiative were followed [42,43]. Specifically, the standard criteria ‘level III’ (putatively characterized compounds), corresponding to compounds that were identified by HR-MS and spectral similarities from databases and literature, was fulfilled for Chem Spider results, while the standard criteria “level IIa” (probable structure) that were based on matching them with literature or library spectrum data, wherein the spectrum-structure match was unambiguous [e.g., tandem MS (MS^2^) profile], was considered for validating mzCloud entries. For more reliable identification, Chem Spider and mzCloud results were visually inspected by the operator, and only those compounds fulfilling some more stringent criteria internally defined were taken into consideration. Specifically, Chem Spider features were refined, taking into account only compounds that showed a mass shift experimental/theoretical ≤3 ppm along with an isotope pattern where the ion precursor together with the M+1 and M+2 isotopes were correctly recognized. On the contrary, mzCloud results were screened on the base of the matching fragmentation spectra of each identified molecule. In particular, only features showing ≥2 most abundant fragments that were correctly recognized were considered.

After identification, each molecule was searched in PubChem database to retrieve the class to which the compound belongs or to obtain any other useful information to elucidate its function.

Finally, the data matrix (acquired both in positive and negative ionization modes) containing the area values that were provided by Compound Discoverer for phenolic compounds features was used for comparative analysis to obtain preliminary information about the likely antioxidant capabilities of green and red lentils.

## 4. Conclusions

The microwave irradiation effect and the solvent effect on the extraction of bioactive compounds from lentil hulls were thoroughly investigated, and the antioxidant potential of the obtained extracts was determined. Our study confirms that the antioxidant content of lentil hulls is higher than in seeds and demonstrates that a higher content of bioactive compounds was recovered through a 5 min extraction with MAE rather than with the conventional solid–liquid extraction. Water proved to be the most efficient extraction solvent in terms of the high levels of recovery of the bioactive and/or antioxidant compounds and having eco-friendly properties. Specifically, the red lentil hull showed a higher antioxidant content than the green lentil variety. Overall, using waste by-products of agri-food pilot centers/industries, an effective and environmentally friendly extraction procedure was herein developed. Finally, these cultivars were for the first time investigated through a metabolite discovery analysis using HPLC separation which was coupled with an Orbitrap-based High-Resolution Mass Spectrometry technique which was followed by an informatic data treatment by means of dedicated software for the compound identification. Therefore, a complex metabolomic workflow was exploited to investigate the phytochemical composition of the lentil extracts and identify the putative beneficial compounds accounting for the observed properties, which are displayed on specific cell lines. These compounds include alkaloids, phenolic compounds and small bioactive peptides which are the basic constituent of healthy diets to prevent the development of chronic diseases.

In conclusion, we developed a novel and totally green extraction process for antioxidant compounds from lentil hulls, both in terms of the extraction technique and the solvent that was used This could pave the way for the industrial recovery of pulse by-products as part of a zero-waste circular economy, although more efforts will be needed to scale up the proposed extraction process. Moreover, we demonstrated that lentil hulls could be considered a powerful sources of phytochemicals and nutritional compounds that are endowed with antioxidant properties. Since the reduction in the oxidative stress in the neuronal cells could prevent neurodegeneration, the results that are reported here suggest that our extracts, in perspective, can play the role of nutraceuticals for the prevention and treatment of neurodegenerative diseases, and more generally, they could enter new food formulations for nutrition and health benefits. These preliminary results prompt our research to further investigate and deepen the studies in this regard.

## Figures and Tables

**Table 1:** Antioxidant activity of seeds and hulls lentil extracts, obtained with different solvents, on SH-SY5Y cells.

					IC_50_ (μg/mL) ^a^	
Entry	Sample	Solvent	Method	Yield (%) ^b^	Antioxidant Activity ^c^	Cytotoxicity ^d^
1	Eston green seed	EtOAc	MAE	0.9	>100	>200
2	Eston green hull	EtOAc	MAE	4.1	87 ± 2	110 ± 3
3	Eston green hull	EtOAc	maceration	4.8	126 ± 5	>200
4	Eston green hull	abs EtOH	MAE	4.5	76 ± 1.3	>200
5	Eston green hull	H_2_O	MAE	13	26 ± 7	>200
6	Crimson red hull	H_2_O	MAE	12	10.1 ± 0.6	>200

^a^ Results were expressed as means ± standard deviation (*n* = 3); 25 μg/mL quercetin (QRC) inhibited ROS production by 89%. ^b^ *Yield*% = (weight of the dry *extract*/weight of the dry plant) × 100. ^c^ Evaluated through the H_2_DCF-DA assay. ^d^ Evaluated through the MTT assay.

**Table 2:** Total phenolic, flavonoid contents, and antioxidant activity of lentil hull aqueous extracts of Eston green and Crimson red varieties that were obtained under microwave irradiation.

Samples	TPC ^a^	TFC ^b^	ABTS ^c^	DPPH ^d^
Eston green	7.88 ± 0.1	0.08 ± 0.01	3.43 ± 0.56	0.53
Crimson red	28.3 ± 0.1	1.89 ± 0.01	39.06 ± 0.73	0.39

^a^ Total phenolic content expressed as mg GAE/g dry weight. Values represent means ± SD (*n* = 3). ^b^ Total flavonoid content expressed as mg CE/100 mg dry weight. Values represent means ± SD (*n* = 3). ^c^ Expressed as mg TE/g dry weight. Values represent means ± SD (*n* = 3). ^d^ IC_50_: radical scavenging activity (concentration expressed in μg/mL necessary for 50% reduction of DPPH radical).

**Table 3:** Summary of the most relevant molecules that were identified in the hulls of Eston green and Crimson red lentils after processing the respective high-resolution mass spectrometry (HRMS) spectra using Compound Discoverer software. The level of identification medium * (level III—putatively characterized) and advanced ** (level IIa—probable structure) were specified for each molecule along with the class, based on its detection in the green and red variety (X: presence; empty cell: absence) and any other relevant information. For uncommon compounds, the proper retention time (RT) which was observed in each variety was reported, while for compounds that were found in both of the varieties, the RT values referred to Eston Green analysis have been reported.

Class	Name	Chemical Formula	DeltaMass [ppm]	Molecular Weight (Da)	Adduct	*m*/*z*	RT (min)	Identification Level	Eston Green	Crimson Red
Alkaloids	Sinapine	C_16_H_23_NO_5_	0	309.15762	[M+H]^+^	310.1649	32.112	**	X	X
	Trigonelline	C_7_H_7_NO_2_	0.05	137.04768	[M–H]^−^	138.055	2.024	**	X	X
Aminoacids and derivatives	2-Phenylglycine	C_8_H_9_NO_2_	−0.47	151.06326	[M+H]^+^	152.0705	7.376	**	X	
	5-Aminolevulinic acid	C_5_H_9_NO_3_	−0.52	131.05817	[M+H]^+^	132.0655	1.931	**	X	
	Asparagine	C_4_H_8_N_2_O_3_	0.56	132.05357	[M+H]^+^	133.0609	1.897	**	X	X
	*cis*-4-Decenoyl carnitine	C_17_H_31_NO_4_	−0.92	313.22502	[M+H]^+^	314.2323	30.397	*	X	
	d-Aspartic acid	C_4_H_7_NO_4_	0.07	133.03752	[M+H]^+^	134.0448	19.36	**	X	
	d-Carnitine	C_7_H_15_NO_3_	−1.14	161.10501	[M+H]^+^	162.1123	1.951	**	X	
	Diaminopimelic acid	C_7_H_14_N_2_O_4_	−0.1	190.09534	[M+H]^+^	191.1026	2.069	**		X
	dl-Arginine	C_6_H_14_N_4_O_2_	0.65	174.11179	[M+H]^+^	175.1191	1.873	**	X	X
	dl-Glutamine	C_5_H_10_N_2_O_3_	−0.27	146.0691	[M+H]^+^	147.0764	2.021	**	X	X
	dl-Tryptophan	C_11_H_12_N_2_O_2_	−2.75	204.08932	[M–H]^−^	203.082	21.55	**	X	X
	d-Serine	C_3_H_7_NO_3_	3.08	105.04292	[M+H]^+^	106.0502	2.865	**		X
	Glutinic acid	C_5_H_4_O_4_	1.21	128.01111	[M+H]^+^	129.0184	4.388	*	X	
	Glycylglycyl-*N*^5^-(phosphonoacetyl)-l-ornithine	C_11_H_21_N_4_O_8_P	2.64	368.11067	[M–H]^−^	367.1034	47.585	*		X
	Indole-3-acetyl-l-aspartic acid	C_14_H_14_N_2_O_5_	1.15	290.09061	[M–H]^−^	289.0833	38.764	**	X	X
	l-2-Aminoadipic acid	C_6_H_11_N O_4_	−0.5	161.06873	[M+H]^+^	162.076	2.833	**	X	
	l-Aspartic acid	C_4_H_7_N O_4_	0.07	133.03752	[M+H]^+^	134.0448	1.913	**	X	
	l-Glutamic acid	C_5_H_9_NO_4_	0.35	147.05321	[M+H]^+^	148.0604	1.966	**	X	X
	l-Kynurenine	C_10_H_12_N_2_O_3_	−0.4	208.08471	[M+H]^+^	209.092	53.014	**	X	
	l-Pyroglutamic acid	C_5_H_7_NO_3_	0.26	129.04263	[M+H]^+^	130.0499	4.146	**	X	
	l-Tyrosine	C_9_H_11_NO_3_	−4.57	181.07307	[M−H]^−^	180.0658	5.941	**	X	
	Muramic acid	C_9_H_17_N O_7_	−0.74	251.10031	[M+H−H_2_O]^+^	234.097	1.966	**		X
	*N*-({(1*R*,2*S*)-2-[(2*Z*)-5-Hydroxy-2-penten-1-yl]-3-oxocyclopentyl}acetyl)-l -isoleucine	C_18_H_29_NO_5_	−0.38	339.20444	[M+H]^+^	340.2115	43.883	*		X
	*N*^2^-[2-(Carboxymethyl)-2-hydroxy-3-methoxy-3-oxopropanoyl]arginine	C_12_H_20_N_4_O_8_	−1.04	348.12775	[M+H]^+^	349.135	3.966	*		X
	*N*^3^,*N*^4^-Dimethyl-l -arginine	C_8_H_18_N_4_O_2_	−0.27	202.14292	[M+H]^+^	203.1502	2.08	**	X	
	*N*^6^,*N*^6^,*N*^6^-Trimethyl-l-lysine	C_9_H_20_N_2_O_2_	−0.02	188.15247	[M+H]^+^	189.1598	1.793	**	X	X
	*N*^6^-Acetyl-l-lysine	C_8_H_16_N_2_O_3_	−0.59	188.11598	[M+H]^+^	189.1233	3.015	**		X
	*N*-Acetyl-dl-glutamic acid	C_7_H_11_NO_5_	−4.18	189.06293	[M−H]^−^	188.0557	4.651	**	X	
	*N*-Acetyl-dl-tryptophan	C_13_H_14_N_2_O_3_	−0.6	246.10029	[M−H]^−^	245.093	47.529	**	X	X
	*N*-Acetyldopamine	C_10_H_13_NO_3_	−0.04	195.08954	[M+H]^+^	196.0968	19.554	**	X	
	*N*-Acetylornithine	C_7_H_14_N_2_O_3_	0.19	174.10048	[M+H]^+^	175.1078	2.429	**	X	X
	*O*-Acetylserine	C_5_H_9_NO_4_	−0.65	147.05306	[M+H]^+^	148.0603	2.837	**		X
	*trans*-3-Indoleacrylic acid	C_11_H_9_NO_2_	0.35	187.06339	[M+H]^+^	188.0707	20.891	**	X	X
Nucleosides/Nucleotides/Nitrogenous bases	2′-Deoxyadenosine	C_10_H_13_N_5_O_3_	−1.1	251.10156	[M+H]^+^	252.1088	7.987	**		X
	5′-Deoxy-5′-(methylsulfinyl)adenosine	C_11_H_15_N_5_O_4_S	−1.36	313.08405	[M+H]^+^	314.0913	6.449	*		X
	Adenine	C_5_H_5_N_5_	−0.14	135.05448	[M+H]^+^	136.0618	7.514	**	X	X
	Adenosine	C_10_H_13_N_5_O_4_	−0.16	267.09671	[M+H]^+^	268.1039	7.457	**	X	X
	Adenosine 5′-monophosphate	C_10_H_14_N_5_O_7_P	−0.54	347.0629	[M+H]^+^	348.07	3.301	**	X	X
	Cytidine	C_9_H_13_N_3_O_5_	−1.1	243.08525	[M+H]^+^	244.0925	3.16	*		X
	Guanine	C_5_H_5_N_5_O	−0.3	151.04936	[M+H]^+^	152.0566	3.178	**	X	X
	Guanosine	C_10_H_13_N_5_O_5_	0.34	283.09177	[M−H]^−^	282.0847	8.841	**	X	
	Hypoxanthine	C_5_H_4_N_4_O	−0.01	136.03851	[M+H]^+^	137.0458	8.598	**	X	X
	Succinyladenosine	C_14_H_17_N_5_O_8_	0.44	383.10788	[M−H]^−^	382.1009	22.063	*	X	
	Thymidine	C_10_H_14_N_2_O_5_	−0.59	242.09013	[M−H]^−^	241.0828	12.561	*		X
	Thymidine 5′-monophosphate	C_10_H_15_N_2_O_8_P	0.35	322.05672	[M−H]^−^	321.0494	5.655	**		X
	UDP-*N*-acetylglucosamine	C_17_H_27_N_3_O_17_P_2_	1.98	607.08277	[M−H]^−^	606.0755	1.98	**	X	X
	Uridine	C_9_H_12_N_2_O_6_	−0.13	244.06951	[M−H]^−^	243.0622	5.204	**	X	X
	Uridine monophosphate (UMP)	C_9_H_13_N_2_O_9_P	0.59	324.03606	[M−H]^−^	323.0288	2.09	**	X	X
	Xanthine	C_5_H_4_N_4_O_2_	−0.51	152.03335	[M+H]^+^	153.0406	11.922	**	X	X
	Xanthosine	C_10_H_12_N_4_O_6_	0.71	284.07589	[M−H]^−^	283.0686	11.276	**	X	X
Phenolic compounds	2-Hydroxycinnamic acid	C_9_H_8_O_3_	−0.28	164.0473	[M+H]^+^	165.0546	46.303	**		X
	4,5-Dicaffeoylquinic acid	C_25_H_24_O_12_	1.39	516.12749	[M−H]^−^	515.1202	52.802	**	X	X
	4-Coumaric acid	C_9_H_8_O_3_	−0.41	164.04728	[M+H]^+^	165.0546	23.362	**		X
	Afzelin	C_21_H_20_O_10_	−0.52	432.10542	[M+H]^+^	433.1127	47.434	**	X	X
	Astragalin	C_21_H_20_O_11_	0.47	448.10077	[M−H]^−^	447.0937	53.359	**		X
	Caffeic acid	C_9_H_8_O_4_	−0.65	180.04214	[M+H]^+^	181.0494	50.345	**	X	
	Catechin	C_15_H_14_O_6_	0.55	290.0792	[M−H]^−^	289.0721	27.964	**	X	X
	Chlorogenic acid	C_16_H_18_O_9_	0.52	354.09527	[M−H]^−^	353.0881	31.332	**	X	
	*cis*-Resveratrol	C_14_H_12_O_3_	−2.01	228.07819	[M+H]^+^	229.0855	48.31	**	X	X
	Elephantorrhizol	C_15_H_14_O_8_	−1.31	322.06844	[M+H]^+^	323.0757	44.924	*	X	
	Esculetin	C_9_H_6_O_4_	−0.66	178.02649	[M+H]^+^	179.0338	42.794	**		X
	Gallocatechin-4beta-ol	C_15_H_14_O_8_	−1.02	322.06854	[M+H]^+^	323.0758	44.976	*		X
	Gentisic acid	C_7_H_6_O_4_	−0.8	154.02649	[M+H−H_2_O]^+^	137.0232	31.442	**		X
	Hyperoside	C_21_H_20_O_12_	−0.93	464.09504	[M+H]^+^	465.1023	52.357	**	X	
	Isovanillic acid	C_8_H_8_O_4_	−0.42	168.04219	[M+H]^+^	169.0495	16.696	**		X
	Kaempferol	C_15_H_10_O_6_	−1.14	286.04741	[M+H]^+^	287.0547	47.431	**	X	X
	Kaempferol 3-(6″-*p*-coumarylgalactoside)	C_30_H_26_O_13_	−1.13	594.13667	[M+H]^+^	595.144	15.879	*		X
	Kaempferol-3-Galactoside-6″-Rhamnoside-3‴-Rhamnoside	C_33_H_40_O_19_	0.14	740.21648	[M−H]^−^	739.2098	52.091	**		X
	Miquelianin	C_21_H_18_O_13_	0.99	478.07521	[M−H]^−^	477.0682	52.087	**	X	X
	myricetin 3-*O*-beta-d-galactopyranoside	C_21_H_20_O_13_	1.24	480.09099	[M−H]^−^	479.0837	50.067	*	X	
	Myricitrin	C_21_H_20_O_12_	1.51	464.09617	[M−H]^−^	463.0889	51.46	**	X	X
	Naringenin	C_15_H_12_O_5_	0.28	272.06855	[M−H]^−^	271.0615	51.598	**	X	
	Naringeninchalcone	C_15_H_12_O_5_	−1.2	272.06815	[M+H]^+^	273.0754	49.806	**		X
	Neochlorogenic acid	C_16_H_18_O_9_	1.27	354.09553	[M−H]^−^	353.0883	35.142	**	X	X
	*N*-Feruloyloctopamine	C_18_H_19_NO_5_	0.03	329.12633	[M−H]^−^	328.1193	52.074	*	X	
	Nictoflorin	C_27_H_30_O_15_	−0.37	594.15825	[M+H]^+^	595.1653	50.122	*		X
	Quercetin	C_15_H_10_O_7_	−1.12	302.04231	[M+H]^+^	303.0496	53.336	**	X	X
	Quercetin-3β-d-glucoside	C_21_H_20_O_12_	1.42	464.09614	[M−H]^−^	463.0889	52.363	**	X	X
	Resorcinol monoacetate	C_8_H_8_O_3_	−0.2	152.04731	[M+H−H_2_O]^+^	135.0441	51.362	**	X	
	Robinin	C_33_H_40_O_19_	−1.28	740.21543	[M+H]^+^	741.2227	52.068	**	X	X
	Rutin	C_27_H_30_O_16_	1.02	610.15401	[M−H]^−^	609.1467	52.435	**		X
	Taxifolin	C_15_H_12_O_7_	−1.17	304.05795	[M+H]^+^	305.0652	51.306	**	X	
Peptides	ala-glu-trp	C_19_H_24_N_4_O_6_	−2.99	404.16838	[M−H]^−^	403.1611	47.923	*		X
	ala-ser-thr-tyr	C_19_H_28_N_4_O_8_	−2.1	440.18979	[M−H]^−^	439.1825	46.227	*		X
	ala-trp-ala-pro	C_22_H_29_N_5_O_5_	−2.03	443.21597	[M−H]^−^	442.2087	45.78	*		X
	alpha-d-Ko	C_8_H_14_O_9_	−1.33	254.06344	[M−H]^−^	253.0562	1.908	*		X
	asn-tyr-tyr	C_22_H_26_N_4_O_7_	−2.51	458.179	[M−H]^−^	457.1717	51.829	*		X
	asp-asp-his-glu	C_19_H_26_N_6_O_11_	0.79	514.16636	[M−H]^−^	513.1591	51.013	*		X
	asp-asp-phe-pro	C_22_H_28_N_4_O_9_	−1.2	492.18504	[M−H]^−^	491.1779	51.002	*	X	X
	asp-glu-glu-thr	C_18_H_28_N_4_O_12_	−0.77	492.16999	[M−H]^−^	491.1623	2.087	*	X	
	cys-glu-pro-asp	C_17_H_26_N_4_O_9_S	−2.97	462.14067	[M+2H]^2+^	232.0776	46.847	*		X
	gln-trp-tyr	C_25_H_29_N_5_O_6_	−1.06	495.21126	[M−H]^−^	494.204	29.916	*	X	
	gln-tyr-ser	C_17_H_24_N_4_O_7_	−2.61	396.16347	[M−H]^−^	395.1562	30.578	*	X	
	gln-tyr-trp	C_25_H_29_N_5_O_6_	−1.0	495.21129	[M−H]^−^	494.204	24.865	*	X	
	glu-asp-phe-ile	C_24_H_34_N_4_O_9_	−1.9	522.23158	[M−H]^−^	521.2243	52.253	*		X
	glu-gly-glu-glu	C_17_H_26_N_4_O_11_	−2.12	462.15883	[M+FA−H]^−^	507.1569	2.059	*	X	
	glu-trp-glu	C_21_H_26_N_4_O_8_	−2.19	462.17405	[M−H]^−^	461.1668	44.783	*		X
	gly-ser-ser-phe	C_17_H_24_N_4_O_7_	−2.68	396.16344	[M−H]^−^	395.1562	33.391	*	X	
	l-Alanyl-l-proline	C_8_H_14_N_2_O_3_	−0.47	186.10035	[M+H]^+^	187.1076	2.091	**	X	
	l-Glutathione (reduced)	C_10_H_17_N_3_O_6_S	0.45	307.08395	[M−H]^−^	306.0767	53.364	**	X	
	phe-ser-tyr-ala	C_24_H_30_N_4_O_7_	−1.4	486.21077	[M−H]^−^	485.2035	45.812	*	X	
	pro-pro-pro-ser	C_18_H_28_N_4_O_6_	−2.98	396.19971	[M−H]^−^	395.1924	46.35	*		X
	thr-ser-ser-tyr	C_19_H_28_N_4_O_9_	−1.22	456.18507	[M−H]^−^	455.1778	15.261	*	X	
	val-trp-leu-glu	C_27_H_39_N_5_O_7_	−2.43	545.28362	[M−H]^−^	544.2767	53.568	*		X
Vitamins and provitamin	Choline	C_5_H_13_NO	3.95	103.10012	[M+H]^+^	104.1074	1.878	**	X	
	Nicotinamide	C_6_H_6_N_2_O	1.44	122.04819	[M+H]^+^	123.0555	3.533	**	X	
	Panthenol	C_9_H_19_NO_4_	−1.86	205.13103	[M−H]^−^	204.1236	14.547	**	X	X
	Pantothenic acid	C_9_H_17_NO_5_	−1.41	219.11036	[M−H]^−^	218.103	14.391	**	X	X

**Table 4:** Bioactive peptides that were found in water extract of Eston green and Crimson red lentil hulls (X: presence; empty cell: absence in the lentil cv.) after search performed in BIOPEP-UWM^TM^ database.

Class	Sequence (Three-Letter Code)	Sequence (One Letter Code)	Molecular Weight (Da)	Eston Green	Crimson Red	ID Bioactive Peptide	Activity	Sequence Bioactive Peptide
Peptides	ala-glu-trp	AEW	404.1684		X	-		
	ala-ser-thr-tyr	ASTY	440.1898		X	-		
	ala-trp-ala-pro	AWAP	443.2160		X	-		
	asn-tyr-tyr	NYY	458.1790		X	7965	Antioxidant	**NYY**
	asp-asp-his-glu	DDHE	514.1664		X	-		
	asp-asp-phe-pro	DDFP	492.1850	X	X	-		
	asp-glu-glu-thr	DEET	492.1700	X		-		
	cys-glu-pro-asp	CEDP	462.1407		X	-		
	gln-trp-tyr	QWY	495.2113	X		-		
	gln-tyr-ser	QYS	396.1635	X		-		
	gln-tyr-trp	QYW	495.2113	X		9197	Antioxidant peptide	FFRSKLLSDGAAAAKGALLP**QYW**
						9198	Alpha-amylase inhibitor	FFRSKLLSDGAAAAKGALLP**QYW**
						9199	Antioxidant peptide	RCMAFLLSDGAAAAQQLLP**QYW**
						9200	Alpha-amylase inhibitor	RCMAFLLSDGAAAAQQLLP**QYW**
	glu-asp-phe-ile	EDFI	522.2316		X	-		
	glu-gly-glu-glu	EGEE	462.1588	X		-		
	glu-trp-glu	EWE	462.1741		X	8237 8238	Antioxidant	AI**EWE**GIESGSVEQA, I**EWE**GIESGSVEQA
	gly-ser-ser-phe	GSSF	396.1634	X		-		
	ala-pro	AP	186.1004	X		3177	Dipeptidyl peptidase IV inhibitor (DPP IV inhibitor)	**AP**
	LL-Glutathione (reduced)	GSH	307.0840	X		9035	ACE inhibitor	**GSH**
						9954	Antioxidant peptide	
	phe-ser-tyr-ala	FSYA	486.2108	X		-		
	pro-pro-pro-ser	PPPS	396.1997		X	3778	Dipeptidyl carboxypeptidase inhibitor	**PPPS**
	thr-ser-ser-tyr	TSSY	456.1851	X		-		
	val-trp-leu-glu	VWLE	545.2836		X	-		
